# Predictors of progression to chronic dialysis in survivors of severe acute kidney injury: a competing risk study

**DOI:** 10.1186/1471-2369-15-114

**Published:** 2014-07-10

**Authors:** Ziv Harel, Chaim M Bell, Stephanie N Dixon, Eric McArthur, Matthew T James, Amit X Garg, Shai Harel, Samuel Silver, Ron Wald

**Affiliations:** 1 Division of Nephrology, St Michael’s Hospital, University of Toronto, 61 Queen Street, 7th floor, M5C 2 T2, ON Toronto, Canada; 2 Department of Medicine and Keenan Research Centre, Li Ka Shing Knowledge Institute of St Michael’s da; 3 Department of Medicine, Mount Sinai Hospital, University of Toronto, Toronto, Canada; 4 Institute of Clinical Evaluative Sciences, Kidney, Dialysis, Transplantation, London, Ontario, Canada; 5 Division of Nephrology, London Health Sciences Centre, University of Western Ontario, London, Canada; 6 Department of Medicine, Division of Nephrology, University of Calgary, Calgary, Alberta, Canada

**Keywords:** Acute kidney injury, Predictors, Chronic dialysis, Competing risk

## Abstract

**Background:**

Survivors of acute kidney injury are at an increased risk of developing irreversible deterioration in kidney function and in some cases, the need for chronic dialysis. We aimed to determine predictors of chronic dialysis and death among survivors of dialysis-requiring acute kidney injury.

**Methods:**

We used linked administrative databases in Ontario, Canada, to identify patients who were discharged from hospital after an episode of acute kidney injury requiring dialysis and remained free of further dialysis for at least 90 days after discharge between 1996 and 2009. Follow-up extended until March 31, 2011. The primary outcome was progression to chronic dialysis. Predictors for this outcome were evaluated using cause-specific Cox proportional hazards models, and a competing risk approach was used to calculate absolute risk.

**Results:**

We identified 4 383 patients with acute kidney injury requiring temporary in-hospital dialysis who survived to discharge. After a mean follow-up of 2.4 years, 356 (8%) patients initiated chronic dialysis and 1475 (34%) died. The cumulative risk of chronic dialysis was 13.5% by the Kaplan-Meier method, and 10.3% using a competing risk approach. After accounting for the competing risk of death, previous nephrology consultation (subdistribution hazard ratio (sHR) 2.03; 95% confidence interval (CI) 1.61-2.58), a history of chronic kidney disease (sHR3.86; 95% CI 2.99-4.98), a higher Charlson comorbidity index score (sHR 1.10; 95% CI 1.05-1.15/per unit) and pre-existing hypertension (sHR 1.82; 95% CI 1.28-2.58) were significantly associated with an increased risk of progression to chronic dialysis.

**Conclusions:**

Among survivors of dialysis-requiring acute kidney injury who initially become dialysis independent, the subsequent need for chronic dialysis is predicted by pre-existing kidney disease, hypertension and global comorbidity. This information can identify patients at high risk of progressive kidney disease who may benefit from closer surveillance after cessation of the acute phase of illness.

## Background

Acute kidney injury (AKI) is a common and serious complication of hospitalization; in severe cases, urgent renal replacement therapy (hereafter referred to as dialysis) is needed to address complications of AKI and to support the patient in overcoming the acute illness. Between 2000 and 2009, the incidence of dialysis requiring AKI (AKI-D) in the United States increased by 10% annually from 222 to 533 cases per million person-years [1]. The increased incidence of AKI will result in greater numbers of patients who are faced with persistent health challenges after the acute phase of their illness has resolved. As a result, acquisition of a better understanding of AKI survivors, who are at increased risk of progressive kidney disease, and death, is of vital public health importance [2-5].

Recognition that survivors of AKI are at high risk of progressive chronic kidney disease (CKD) spurred the Kidney Disease Improving Global Outcomes (KDIGO) AKI guidelines to recommended that kidney function should be evaluated 3 months after an AKI episode to establish the presence and extent of chronic kidney disease [6] However, others have advocated that Nephrology follow-up occur for all patients with severe AKI [7]. With almost 1 in 5 adults worldwide experiencing an episode of AKI during a hospitalization, two percent of whom require dialysis, such a broad referral strategy may be impractical [8]. Identifying AKI-D survivors at the greatest risk of progressive CKD would potentially lead to the targeted follow-up for individuals who are most likely to realize a benefit. Accordingly, in a cohort of AKI-D survivors, we performed a study to determine the predictors of permanent maintenance dialysis and secondarily, death.

## Methods

### Design and participants

We conducted a retrospective study using population-wide linked administrative health databases for all of Ontario, Canada. We identified Ontario residents aged 19 years and older admitted to an acute care hospital between April 1, 1996 and March 31, 2009, for whom length of stay was less than 180 days. We focused on individuals who had a diagnosis of AKI and a claim for acute dialysis during a hospital stay, and lived for at least 90 days after hospital discharge without re-initiation of dialysis or rehospitalization during this 90-day period. We excluded individuals with a previous diagnosis of AKI, receipt of a kidney transplant, or any form of dialysis in the 5-year period preceding the index hospitalization. Ontario is Canada’s most populous province, and all residents are covered by a government-funded universal healthcare program. No informed written consent was obtained from each individual included in our study as administrative data was used. The study was approved by the research ethics board of Sunnybrook Hospital.

### Data sources

The study was completed using linked records from six administrative health care databases using encrypted unique identifiers. Hospitalization records were obtained from the Canadian Institute for Health Information Discharge Abstract Database (DAD), which was used to identify an acute hospitalization, the presence of AKI, as well as inpatient comorbidities and procedural information on the basis of codes from the International Classification of Diseases (ICD 9th or 10th Revision). The Ontario Health Insurance Plan (OHIP) database provided information on physician claims for inpatient and outpatient services, including dialysis. (Additional file 1) The Ontario Registered Persons Database contained basic demographic information for each eligible individual including date of death. Income quintile was based on postal codes for residence area as outlined in the 2001 Statistics Canada Census. Permission to use these databases was granted by the research ethics board of Sunnybrook Hospital.

### Candidate variable definitions

Candidate variables were selected based on review of the literature and clinical relevance and encompassed demographics, comorbidities, features of the index hospitalization, and markers of health services utilization. (Additional file 1) All demographic, comorbidity, and health care utilization covariates were ascertained from 5 years preceding the hospital discharge date except chronic kidney disease which had a lookback period of 5 years preceding the hospital admission date. Similar to previous studies, we included both the Charlson comorbidity index score and the individual components of the score in our model, including chronic kidney disease. The Charlson comorbidity score is a validated method for estimating the risk of death from comorbid disease, and has been used in multiple studies [9]. Each comorbid condition included in the Charlson index is assigned a score of 1, 2, 3, or 6, depending on the risk of dying associated with each condition [9]. The individual scores are summed to provide a total comorbidity score (Charlson comorbidity score) to predict mortality. A standard algorithm proposed by Quan et al. was used to define the Charlson comorbidity score using the DAD [10]. In line with previous studies, we defined CKD using administrative coding (see Additional file 2 for codes), which is highly specific but poorly sensitive (median sensitivity 41%; median specificity 98%) [4,11].

### Outcomes

The period of follow-up commenced at day 90 following discharge for all patients. This period is in line with that used in other AKI follow-up studies in order to mitigate bias that may be attributed to the high mortality rate after discharge among patients with AKI who require temporary renal replacement therapy likely as a result of the underlying illness that mediated the AKI [11-13]. The main outcome was chronic dialysis, which was defined as the receipt of dialysis for at least 90 days [4]. This definition is widely accepted and has been used in multiple studies of AKI [4,5,11]. The date of the first dialysis claim was considered to be the date of initiation of dialysis. The secondary outcome was all-cause mortality.

### Statistical analyses

Baseline characteristics of the study participants were summarized using descriptive statistics. Continuous variables were expressed as mean (SD) or median (interquartile range [IQR]) and compared using the unpaired t-test or Kruskal-Wallis test, respectively. Categorical variables were expressed as a percentage and compared using a χ^2^ test or Fisher’s exact test.

#### Cause specific Cox hazard model and competing risk analysis

The associations between baseline covariates and outcomes were assessed using Cox proportional hazards regression. For the primary outcome of chronic dialysis, subjects were censored in the event of death or at the conclusion of follow-up, March 31, 2011. For the all-cause mortality outcome, subjects were censored at the conclusion of the follow-up, March 31, 2011.

Candidate variables that were associated with the outcome of interest (P ≤ .10) on univariate analysis and those that were deemed to be clinically important regardless of statistical significance were included as potential covariates in the multivariable Cox proportional hazards models. The presence of collinearity within the models was examined by evaluation of variance inflation factors. Time-dependent variables were created to evaluate whether the proportional hazards assumption was met. In cases where the proportional hazards assumption was not met, the candidate variable was modeled as a time-dependent covariate.

As death was a competing risk for the receipt of chronic dialysis, using the method of Fine and Gray, we also developed a semiparametric Cox proportional hazards model that estimated the risk of chronic dialysis initiation with death considered as a competing risk [14].

The standard Cox model and the competing risk approach differ in the way absolute risk predictions are calculated. Whereas predictions from the standard Cox model depend only on the cause-specific hazard of the event of interest (receipt of chronic dialysis) and thus overestimate absolute risk in the presence of a competing event (death), the competing risk approach consists of developing a cause-specific hazards models for both the event of interest (the receipt of chronic dialysis) and the competing event (death) separately, and then combining them according to the cumulative incidence function [15].

#### Model performance

Model discrimination for each of the cause-specific Cox hazard models (chronic dialysis and all-cause mortality) was assessed for discrimination using the Harrell global C-statistic.

Baseline comparisons and development of cause-specific Cox proportional hazards models were performed using SAS version 9.1 for UNIX (SAS Institute, Cary, NC), and the competing risk model was developed using R version 2.9.1 (R Foundation for Statistical Computing, Vienna, Austria).

## Results

### Characteristics of study participants

**Figure 1:**
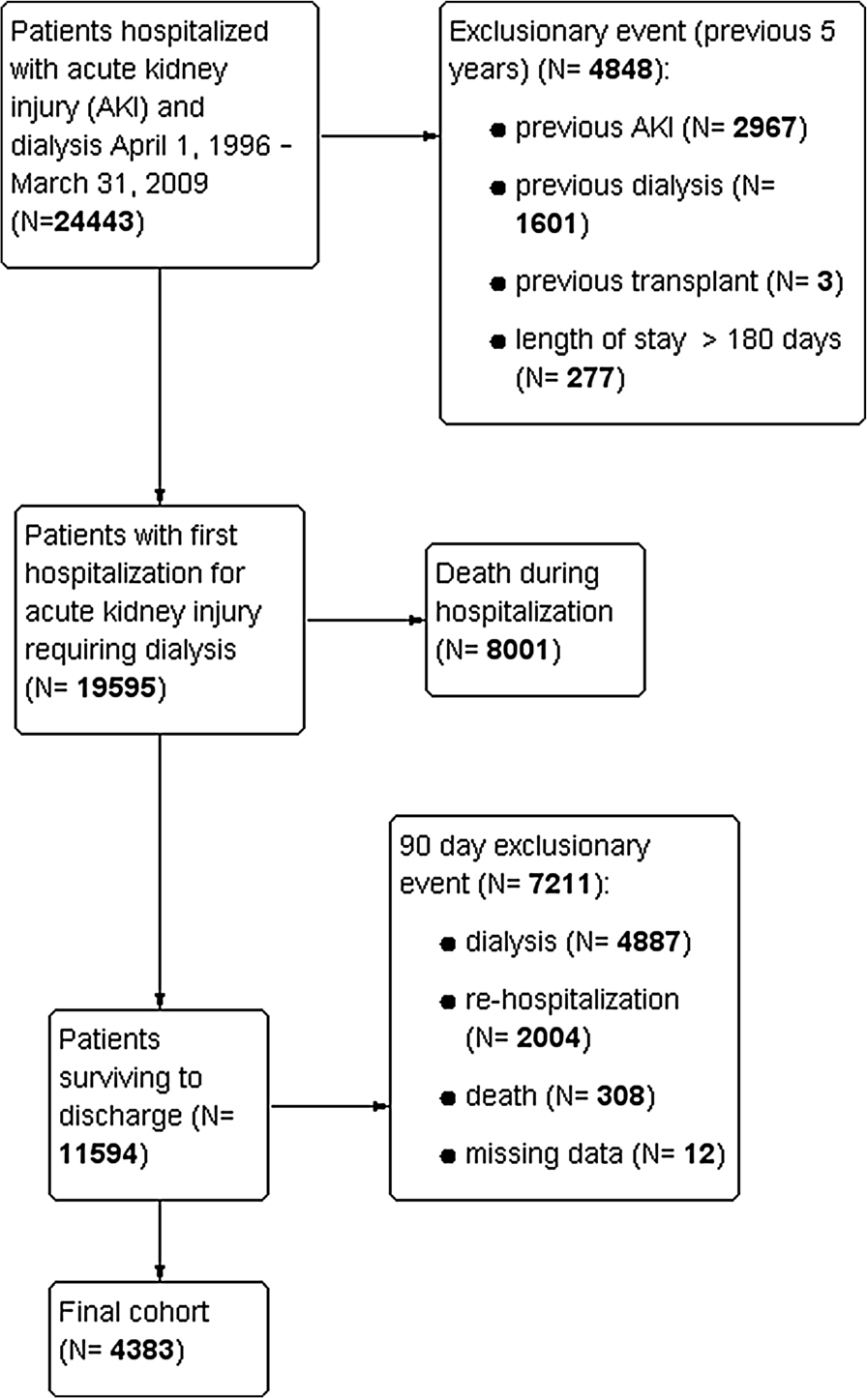
Cohort creation.

We identified 4 383 patients with a previous episode of AKI-D who survived to 90 days following hospital discharge with no reinitiation of dialysis. (Figure 1) Mean age was 61 (±16) years, 60% were male and 28% had seen a nephrologist prior to the index hospitalization on which the AKI-D occurred. The median Charlson comorbidity score was 3 (IQR 1–4). The prevalence of pre-existing coronary artery disease, diabetes, chronic kidney disease, and cancer were 49%, 44%, 17.2%, and 39% respectively.

Patients who progressed to chronic dialysis (n = 356, 8.1% of cohort ) were older (64 vs. 61 years in patients with no progression to chronic dialysis, p < 0.01), had a higher likelihood of previous visit to a nephrologist (60% vs. 25%, p < 0.01) and cardiologist (61% vs. 46%, p < 0.01), and had a higher burden of comorbid disease (including cardiac disease (59% vs. 48%, p < 0.01), congestive heart failure (54% vs. 36%, p < 0.01), diabetes (61% vs. 43%, p < 0.01), hypertension (87% vs. 68%, p < 0.01), peripheral vascular disease (6% vs. 3%, p < 0.01), cancer (43% vs. 39%, p < 0.01), and chronic kidney disease (57% vs. 14%, p < 0.01)) than those who did not progress to chronic dialysis (n = 4,027). On the other hand, individuals who progressed to chronic dialysis were significantly less likely to require mechanical ventilation (46% vs. 59%, p < 0.01), or have a diagnosis of sepsis during their index admission (14% vs. 20%, p < 0.01) (Table 1).

**Table 1:** Baseline characteristics of the cohort stratified by progression to chronic dialysis

**Variable**	**Chronic dialysis**	**No chronic dialysis**	**p-value**
	**N = 356**	**N = 4,027**	
***Demographics and health utilization***			
Age, mean (SD)	64 (±14)	61 (±17)	<0.01
Female gender, n (%)	143 (40)	1 629 (41)	0.92
Rural locale	53 (15)	548 (14)	0.58
Pre-admission nephrology consultation, n (%)	213 (60)	1 004 (25)	<0.01
Pre-admission cardiology consultation, n (%)	216 (61)	1 835 (46)	<0.01
***Comorbid disease***			
Charlson comorbidity score, median (IQR)	4 (2–6)	2 (1–4)	<0.01
Coronary artery disease, n (%)	209 (59)	1 933 (48)	<0.01
Congestive heart failure, n (%)	191 (54)	1 439 (36)	<0.01
Cerebrovascular disease, n (%)	63 (18)	713 (18)	1
Diabetes, n (%)	218 (61)	1 712 (43)	<0.01
Malignancy, n (%)	153 (43)	1 560 (39)	0.12
Liver disease, n (%)	27 (8)	644 (16)	<0.01
Peripheral vascular disease, n (%)	21 (6)	115 (3)	<0.01
Chronic kidney disease, n (%)	204 (57)	551 (14)	<0.01
Hypertension, n (%)	309 (87)	2 738 (68)	<0.01
Dementia, n (%)	25 (7)	334 (8)	0.40
Proteinuria, n (%)	9 (3)	13 (0.3)	<0.01
Hematuria, n (%)	125 (35)	1 060 (26)	<0.01
***Conditions during index hospitalization***			
Sepsis, n (%)	49 (14)	807 (20)	<0.01
Aortic aneurysm repair, n (%)	10 (3)	155 (4)	0.32
Cardiac surgery, n (%)	10 (3)	155 (4)	0.32
Mechanical ventilation, n (%)	163 (46)	2 209 (55)	<0.01
***Hospital type***			
Teaching Hospital, n (%)	173 (49)	1 980 (49)	0.84

^a^In the 5 years preceding hospital admission.

^b^In the 5 years preceding the hospital discharge date except chronic kidney disease which had a lookback period of 5 years preceding the hospital admission date.

Patients who died during the study follow-up (n = 1,475, 33.7% of total cohort) were older (68 vs. 56 years, p <0.01), more likely to have seen a nephrologist (30% vs. 22%, p < 0.01), and had a higher prevalence of pre-exisiting comorbidities (including cardiac disease (57% vs. 43%, p < 0.01), congestive heart failure (49% vs. 28%, p < 0.01), diabetes (48% vs. 40%, p < 0.01), cancer (49% vs. 33%, p < 0.01), chronic kidney disease (19% vs. 10%, p < 0.01), and hypertension (76% vs. 63%, p < 0.01) (Table 2).

**Table 2:** Baseline characteristics of the cohort stratified by death during the follow-up period

**Variable**	**Death**	**Alive**	**p-value**
	**N = 1,475**	**N = 2.908**	
***Demographics and health utilization***			
Age, mean (SD)	68 (±14)	56 (±17)	<0.01
Female gender, n (%)	589 (40)	1 040 (41)	0.61
Rural locale, n (%)	206 (14)	395 (14)	0.73
Pre-admission nephrology consultation, n (%)^a^	441 (30)	563 (22)	<0.01
Pre-admission cardiology consultation, n (%)^a^	750 (51)	1 085 (43)	<0.01
***Comorbid disease***^***b***^			
Charlson comorbidity score, median (IQR)	4 (2–5)	2 (0–3)	<0.01
Coronary artery disease, n (%)	842 (57)	1 091 (43)	<0001
Congestive heart failure, n (%)	718 (49)	721 (28)	<0.01
Cerebrovascular disease, n (%)	349 (24)	364 (14)	<0.01
Diabetes, n (%)	700 (48)	1012 (40)	0.08
Malignancy, n (%)	715 (49)	845 (33)	<0.01
Liver disease, n (%)	214 (15)	430 (17)	0.05
Peripheral vascular disease, n (%)	82 (6)	33 (1)	<0.01
Chronic kidney disease, n (%)	286 (19)	265 (10)	<0.01
Hypertension, n (%)	1 122 (76)	1616 (63)	<0.01
Dementia, n (%)	182 (12)	152 (6)	<0. 01
Hematuria, n (%)	457 (31)	603 (24)	0.5
Proteinuria, n (%)	6 (0.4)	7 (03)	<0.01
***Conditions during index hospitalization***			
Sepsis, n (%)	280 (19)	527 (21)	0.2
Aortic aneurysm repair, n (%)	69 (5)	86 (3)	0.04
Cardiac surgery, n (%)	152 (10)	320 (13)	0.03
Mechanical ventilation, n (%)	705 (48)	1 504 (59)	<0.01
***Hospital type***			
Teaching hospital, n (%)	746 (48)	1 406 (51)	0.15

^a^In the 5 years preceding hospital admission.

^b^In the 5 years preceding the hospital discharge date except chronic kidney disease which had a lookback period of 5 years preceding the hospital admission date.

### Outcomes

**Figure 2:**
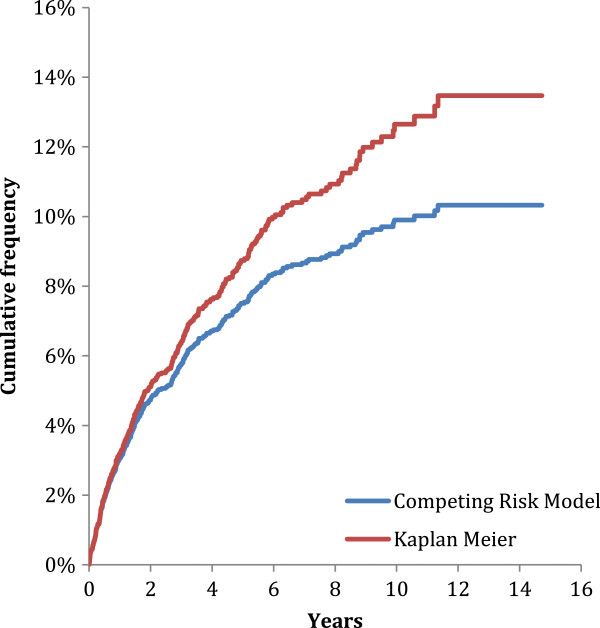
Cumulative incidence of chronic dialysis initiation by model.

After a mean follow-up of 2.4 years, the cumulative incidence of chronic dialysis was 13.5% by the Kaplan-Meier method, and 10.3% using the competing risk approach. (Figure 2) The cumulative incidence of all-cause mortality was 54.2%, after a mean follow-up of 3.0 years, and 1475 patients died prior to the initiation of chronic dialysis therapy.

### Predictors of chronic dialysis

Previous nephrology consultation (adjusted hazard ratio (aHR) 2.12, 95% CI 1.67-2.70), Charlson comorbidity score (aHR 1.17; 95% CI 1.11-1.24 per unit increase), a history of chronic kidney disease (aHR 4.04, 95% CI 3.14-5.2), and pre-existing hypertension (aHR 1.70; 95% CI 1.21-2.37) were significantly associated with an increased risk of chronic dialysis. Prior liver disease (aHR 0.42; 95% CI 0.28-0.63) and the receipt of mechanical ventilation on the index hospitalization (aHR 0.78; 95% CI 0.62-0.97) were inversely associated with chronic dialysis. (Table 3) The c-statistics for the chronic dialysis model using the cause-specific Cox approach was 0.66 (95% CI 0.62-0.71).

**Table 3:** Multivariable-adjusted cause-specific hazards ratio for chronic dialysis and all-cause mortality

**Variables**	**Hazard ratios (95% CI)**	
	**Chronic dialysis**	**All-cause mortality**
Age	0.99 (0.99-1.00)	1.03 (1.02-1.04)*
Female gender	0.95 (0.76-1.18)	0.91(0.82-1.01)
Previous nephrology consultation	2.12 (1.67-2.70)*	1.02 (0.91-1.16)
Charlson comorbidity index	1.17 (1.11-1.24)*	1.21 (1.18-1.23)*
Coronary artery disease	0.86 (0.67-1.10)	0.92 (0.82-1.03)
Congestive heart failure	1.22 (0.96-1.56)	1.18 (1.05-1.33)*
Diabetes	1.15 (0.0.91-1.46)	0.87 (0.77-0.97)*
Liver disease	0.42 (0.28-0.63)*	1.07 (0.92-1.25)
Peripheral vascular disease	1.03 (0.66-1.62)	1.32 (1.05-1.66)
Chronic kidney disease	4.04 (3.14-5.20)*	1.01 (0.87-1.17)
Hypertension	1.70 (1.21-2.37)*	0.93 (0.82-1.06)
Dementia	-----	1.30 (1.10-1.50)*
Hematuria	1.10 (0.88-1.37)	1.10 (0.99-1.23)
Proteinuria	1.85 (0.94-3.67)	-----
Sepsis	0.84 (0.62-1.15)	-----
Receipt of mechanical ventilation during index admission	0.78 (0.62-0.97)*	0. 79 (0.71-0.87)*

*p < 0.05.

### Predictors of mortality

Older age (aHR 1.03; 95% CI 1.02-1.04 per year), Charlson comorbidity score (aHR 1.21; 95% CI 1.18-1.23 per unit increase in the score), heart failure (aHR 1.18; 95% CI 1.05-1.33), peripheral vascular disease (aHR 1.32; 95% CI 1.05-1.66), and dementia (aHR 1.30; 95% CI 1.10-1.50) were significantly associated with a higher risk of death. Pre-existing diabetes (aHR 0.87; 95% CI 0.77-0.97), and the receipt of mechanical ventilation during the index hospitalization (aHR 0.79; 95% CI 0.71-0.87), were associated with a lower risk of death. (Table 3) The c-statistics for the all-cause mortality models using the cause-specific Cox approach was 0.71 (95% CI 0.67-0.76).

### Competing risk approach

In multivariable analysis using a Fine and Gray regression model that treated death as a competing event, a history of chronic kidney disease (sHR 3.86; 95% CI 2.99-4.98), a history of previous nephrology consultation (subdistribution hazard ratio (sHR) 2.03, 95% CI 1.61-2.58), Charlson comorbidity index score (sHR 1.10; 95% CI 1.05-1.15), and hypertension (sHR 1.82; 95% CI 1.28- 2.58) were significantly associated with the need for chronic dialysis initiation. The presence of liver disease (sHR 0.43; 95% CI 0.29-0.64) was significantly associated with a lower risk for the initiation of chronic dialysis (Table 4).

**Table 4:** Multivariable model for initiation of chronic dialysis using the competing risk approach

**Variable**	**Subdistribution hazard ratios (95% CI)**
Age	0.99 (0.98-1.00)
Female gender	0.95 (0.77-1.18)
Previous nephrology consultation	2.03(1.61-2.58)*
Charlson comorbidity index	1.10 (1.05-1.15)*
Coronary artery disease	0.87 (0.67-1.13)
Congestive heart failure	1.21 (0.94-1.56)
Diabetes	1.18 (0.93-1.51)
Liver disease	0.43 (0.29-0.64)*
Peripheral vascular disease	0.90 (0.56-1.46)
Chronic kidney disease	3.86 (2.99-4.98)*
Hypertension	1.82 (1.28-2.58)*
Hematuria	1.06 (0.84-1.33)
Proteinuria	2.13 (0.92-4.97)
Sepsis	0.83 (0.61-1.13)
Receipt of mechanical ventilation during index admission	0.85 (0.68-1.05)

*p < 0.05.

## Discussion

Escalating comorbidity, pre-existing kidney disease, as reflected by a diagnosis of CKD or a visit to a nephrologist, and pre-existing hypertension predicted progression to chronic dialysis in a cohort of patients who survived an episode of dialysis-requiring AKI. Similarly, increasing age, a higher Charlson comorbidity score; the presence of heart failure, diabetes and dementia were predictive of post-AKI mortality.

Although the link between AKI and subsequent CKD has been extensively described, patients with the most severe form of AKI who require acute dialysis are the sub-population of patients with the highest risk of progression [16]. However, we found that only 10% of these patients progress to chronic dialysis thus highlighting the need to identify risk factors for such progression in order to target potential interventions to those who are most likely to benefit. Moreover, due to our study design, patients with severe AKI who did not recover renal function or those who initiated chronic dialysis within 90 days of discharge were excluded from our cohort; and therefore, the contribution of AKI to the progression to chronic dialysis is likely higher than our data suggests.

Similar to our report, studies by Stads et al. [17], Ishani et al. [18], and Lo et al. [3] have demonstrated the strong association between pre-existing CKD and the need for subsequent chronic dialysis. It is not surprising that pre-existing CKD is one of the most important predictors modifying the relationship between AKI and progressive CKD as has been demonstrated in multiple studies [19-22]. The impact of even a modest acute insult to already compromised kidneys may be devastating. Although the biological mechanism linking CKD progression after AKI has not fully been elucidated, it has been postulated that the combination of acute endothelial injury leading to vascular dropout, and nephron loss followed by glomerular hypertrophy and the development of fibrosis may all play a role [23,24]. This may be more pronounced in patients with underlying CKD who may already possess a limited renal reserve, such that an episode of AKI may tip them into the need for chronic dialysis more easily than those without a background history of renal disease. Recognizing the limited performance of CKD diagnosis codes, we enhanced our capture of patients with underlying kidney disease by examining prior visits to a nephrologist, which we postulated as a reasonable surrogate for kidney disease. Both prior visits to a nephrologist and/or diagnosed CKD together capture the notion that patients who develop severe AKI with already-compromised kidney function are at substantially higher of accelerated and irreversible kidney dysfunction once the acute illness resolves.

As demonstrated by Chawla et al., we found that hypertension was another important predictor of chronic dialysis among AKI-D survivors [25]. Systemic hypertension is a potent contributor to the development of arteriosclerosis, tubulointerstitial fibrosis and glomerulosclerosis, all of which may hasten the decline in kidney function [26,27]. Patients with a history of hypertension may have underlying unrecognized CKD, despite having a “normal” serum creatinine level [28,29]. Subsequent renal injury imparted by an episode of AKI may not only unmask this underlying damage, but also lead to its acceleration.

The inverse association between liver disease and chronic dialysis was surprising given that these patients are prone to ongoing renal injury from changes in autoregulation due to arteriolar vasodilation and neuroendocrine changes associated with decompensated liver disease [30]. Perhaps, this group of patients underwent acute dialysis during their hospitalization as a bridge toward subsequent liver transplantation. Indeed, serum creatinine is a component of the Model for End-Stage Liver Disease (MELD) score which is used for organ allocation in patients with liver disease [31]. Severe AKI associated with liver disease is associated with extremely high mortality [32]. We speculate that the few individuals with liver disease who survived a hospitalization associated with AKI-D had a relatively lower burden of comorbidity. By the same token, the apparent “protective” association between mechanical ventilation on the index hospitalization and both chronic dialysis and all-cause mortality suggests that the sickest patients who received mechanical ventilation likely died during the hospitalization or shortly thereafter. Those fortunate to survive to 90 days following discharge were a selected group who by virtue of surviving the acute phase of their illness, were destined to have better outcomes.

Since the risk of death after an episode of AKI-D far outstrips that of chronic dialysis, predicting this outcome is also of vital clinical interest. As such, our study also delineated a number of predictors of post-AKI mortality including increasing age, a higher Charlson comorbidity score; the presence of heart failure, diabetes and dementia; as well as an inverse relationship with the receipt of mechanical ventilation. In line with previous work by Stads et al., preexisting CKD was strongly associated with death, which may relate to the link between CKD and cardiovascular disease [17,33].

Our study has several strengths. Most notably, it is the first to determine the predictors of initiating chronic dialysis in a cohort of survivors of severe AKI using a competing risk approach. Although previous studies of AKI survivors have reported similar predictors, there are notable differences in methodology, case-mix and outcome ascertainment between these studies and ours [3,5,18,25,34]. Our use of a competing risk approach, may have avoided overestimating the cumulative incidence of the progression to chronic dialysis in survivors of severe AKI [35], and also gave us more robust effect estimates for our predictors [35]. Finally, our study was conducted in a jurisdiction with universal access to medical services and our databases reflect the entire population, maximizing generalizability. Though non-dialysis requiring CKD following AKI - is common and potentially problematic, we focused our attention on the extreme form of CKD, chronic dialysis, which has the most profound implications for quality of life and survival [2].

Our results have several potential implications. First, the identification of novel predictors of chronic dialysis in survivors of severe AKI may help improve the follow-up of these patients by selectively referring to nephrologists those patients who possess characteristics which place them at a high risk of progression. There is compelling data regarding the positive role that nephrologists may play in survivors of severe AKI; however, only a small proportion of severe AKI survivors are referred to a nephrologist for follow-up [2,11]. This may be related to limited access to nephrologists or an under-appreciation of the risk for progressive CKD following AKI. Alternatively, a select number of patients with pre-existing chronic kidney disease may already be followed by a nephrologist prior to their episode of AKI, and this continuity in care persists after the episode; thereby, obviating the requirement of a new referral. Second, knowledge of predictors of chronic dialysis may allow for the creation of a predictive model that can stratify AKI survivors at highest risk for progression to CKD for inclusion in clinical trials.

Several limitations merit consideration. First, our reliance on administrative codes for diagnoses and procedures limited our ability to better characterize the cohort and incorporate potentially vital data (eg, severity of acute illness, laboratory data) in to our models. In particular, the absence of pre-hospitalization laboratory data, most notable serum creatinine to estimate the glomerular filtration rate and information on proteinuria, is an important limitation [22,25]. As an alternative, we could only describe CKD using administrative coding, which is highly specific but poorly sensitive (median sensitivity 41%; median specificity 98%) [36], and by the surrogate of prior visits with a nephrologist which is an important shortcoming. Moreover, the administrative databases that we used did not contain data (laboratory and clinical data), which would have allowed us to determine illness severity (e.g., SOFA and APACHE scores). As these scores are useful predictors of outcome failure to account for them in our propensity score may have also contributed to confounding. Our inability to access preadmission laboratory data also precluded us from conducting more rigorous analyses with more robust measures of chronic kidney disease. Similarly, we had no way of evaluating the extent of kidney function recovery at the conclusion of the acute illness. Compromised residual kidney function following an episode of acute kidney injury is likely an important predictor of progression to chronic dialysis. Our definition for the receipt of chronic dialysis may have misclassified individuals who truly developed end-stage renal disease, but who died in the first 90 days after commencing chronic dialysis. However, our definition for the receipt of chronic dialysis is widely accepted, and has been used in multiple studies [5,11]. In addition, while it is interesting that greater global comorbidity, as expressed via the Charlson score, was associated with chronic dialysis and death, we could not identify which aspect(s) of the multicomponent Charlson score was most important. Ultimately, our predictive models showed limited discriminatory capacity suggesting that several factors that our datasets could not capture could have been relevant in anticipating important clinical outcomes following an episode of AKI. Moreover, we only examined a cohort of individuals who experienced the most severe form of AKI. In this regard, our findings may not be generalizable to individuals experiencing less severe yet much more common forms of AKI.

## Conclusions

Using a competing risk model, we have demonstrated important predictors of progression to chronic dialysis in survivors of a hospitalization complicated by AKI requiring dialysis. Greater appreciation of the link between AKI and CKD is appropriately accompanied by calls for greater nephrology follow-up for AKI survivors. Our results identify patients with characteristics that associate with a high risk of kidney disease progression and death who should be targeted for closer post-discharge follow-up. Further studies to develop and validate a more robust predictive model, will help optimize outcomes of AKI survivors.

## Competing interests

The authors declare that they have no competing interests.

## Authors’ contributions

ZH, and RW were involved in the study concept, design and coordination, data analysis, and helped to draft the manuscript. SH, AXG, MTJ, CMB, SND, EM, and SS were involved in the analysis of the data, and helped to draft the manuscript. All authors read and approved the final manuscript.

## Pre-publication history

The pre-publication history for this paper can be accessed here:

http://www.biomedcentral.com/1471-2369/15/114/prepub

## Supplementary Material

Additional file 1 Candidate covariates. Click here for file

Additional file 2 Diagnostic codes used in the study. Click here for file
