# Supplementary material for: Predictors of progression to chronic dialysis in survivors of severe acute kidney injury: a competing risk study
Source: BMC Nephrol. 2014 Jul 10;15:114. doi: 10.1186/1471-2369-15-114 (PMC4105112; doi:10.1186/1471-2369-15-114)
Supplement: Additional file 1 — Candidate covariates. [file 1471-2369-15-114-S1.doc]

**Additional file 1:** Candidate Covariates

| **Characteristic** |  |
| --- | --- |
| *Demographics*  Age, mean (SD), y  Female gender  Rural residence    *Health utilization^a^*  Number of cardiology visits  Number of nephrology visits | *Procedure or condition during index hospitalization*  Sepsis  Cardiac surgery  Abdominal aortic aneurysm repair  Mechanical ventilation ^c^ |
|
|
|
| *Hospital type*  Teaching hospital |  |
| *Comorbid disease^b^*  Coronary artery disease§  Congestive heart failure  Cerebrovascular disease  Diabetes  Maligancy  Liver disease  Peripheral vascular disease¶  Chronic kidney disease  Hypertension  Hematuria  Proteinuria  Charlson comorbidity index, mean (SD), score |  |

§Myocardial infarction or coronary artery bypass or angiography or percutaneous coronary intervention; ¶Peripheral vascular disease or procedure for peripheral vascular disease;

^a^ In the 5 years preceding hospital discharge date.

^b^ In the 5 years preceding the hospital discharge date except chronic kidney disease which had a lookback period of 5 years preceding the hospital admission date.

^c^ The receipt of mechanical ventilation was used as a surrogate for admission to a critical care unit.^1^

Reference List

(1) Scales DC, Guan J, Martin CM, Redelmeier DA. Administrative data accurately identified intensive care unit admissions in Ontario. *J Clin Epidemiol* 2006;59:802-807.
