# Supplementary material for: Predictors of progression to chronic dialysis in survivors of severe acute kidney injury: a competing risk study
Source: BMC Nephrol. 2014 Jul 10;15:114. doi: 10.1186/1471-2369-15-114 (PMC4105112; doi:10.1186/1471-2369-15-114)
Supplement: Additional file 2 — Diagnostic codes used in the study. [file 1471-2369-15-114-S2.docx]

Additional file 2: Diagnostic Codes used in the study.

|  | **Condition/Disease** | **ICD-9** | **ICD-10** | **Diagnostic type** | **Procedure Codes**  **OHIP, CCI, CCP** |
| --- | --- | --- | --- | --- | --- |
| **AKI RRT survivors cohort** | - Age ≥ 18 years - No codes for AKI or any form of dialysis in preceding five yrs - Hospital admission with AKI - Dialysis during hospitalization - alive at 90 days post-discharge - no dialysis during the 90-day period after discharge - **no rehospitalization for 90 days following discharge** | 584.5-584.9  **669.3, 958.5 634.3, 635.3,**  **636.3, 637.3**  **638.3, 639.3** | N17.0-N17.9, **O08.4, T79.5, O90.4** | all | R849, R850, G323, G325, G326, G330, G331, G332, G860, G333, G083, G091, G085, G295, G082, G090, G092, G093, G094, G861, G862, G863, G864, G865, G866, G294, G095, G096  CCP: 51.95, 66.98  CCI:  1PZ21HQBR,  1PZ21HPD4 |
| **Mechanical Ventilation** | - describing a patient’s receipt of mechanical ventilation on an admission of interest (used for matching of cohorts to non-exposed individuals) |  |  |  | G557, G558, G559, G405, G406, G407 |
| **Chronic Kidney Disease** | - **the presence of this condition for any inpatient or outpatient encounter in 5 years preceding date of admission for index hospitalization** | 016.0**x**, 095.4, 250.4**x**, 274.1**x**, 403.xx, 404.xx, **405.01, 405.11, 405.91,** 440.1, 446.21, 581.xx, 582.xx, 583.xx, 585.x, 586, 587.x, **588.0**, **588.8x**, **588.9**, 589.x, **590.0x**, **593.7x**, 791.0, 794.4 | A18.1, N29.0, E10.20, E10.21, E11.20, E11.21, M10.39, I12.0, I12.9, I13.0, I13.1, I13.2, I13.9, **I15.0**, I70.1, M31.0, N03.x, N04.x, N05.x, **N06.x**, **N07.x**, **N08.x**, **N11.x, N12**, **N13.7**, **N13.8, N13.9, N14.x**, **N15.x**, **N16.x**, **N18.0**, **N18.8**, **N18.9x**, N18.90, N18.91, N19, **N25.0, N25.8, N25.9**, N26, N27.x, R80, R94.4 | **all** | **403, 581, 582, 585, 586** |
| **Cardiac surgery** | - includes all cardiac surgery except percutaneous insertions, pacemakers, ICDs - for subcategorization of patients with AKI-RRT on index admission |  |  |  | E646-647 E650-652, E656, E658, E660-661, E670-671 E682, M134,M137, R700, R709-714, R715-718, R720-730, R722-738, R741-743, R746-749, R755, R758-759, R762, R768-774, R870, R874, R920-930, R863, R876 |
| **Aortic aneurysm (non-ruptured)** | - surgical procedures for unruptured aortic (thoracic and abdominal) aneurysms - for subcategorization of patients with AKI-RRT on index admission |  |  |  | R799-803, R816-817, R875 (EVAR) |
| **Sepsis** | - for subcategorization of patients with AKI-RRT on index admission (based on criteria of Martin et. al. NEJM) | 003·1, 036·2 and  038·00–038·90 | A082.1, A39.4, A40.3, A40.9, A41.2, A41.3, A41.4, A41.51, A41.52, A41.58, A41.8, A41.9 | all |  |
| **Nephrologist** | 2) A nephrologist is a physician who, during the study accrual period, had both a) AND b):  a) billed an **A135 code** ≥ 50 times from Jan 1^st^, 1991 to end of accrual period (can be same patient)  b) billed renal dialysis code ( in green to the right; excluded Continuous Renal Replacement Therapy from this list of codes”) ≥50 times from Jan 1^st^, 1991 to end of accrual period.  time period |  |  |  | G323, G325, G326, G860, G330, G331,G332, G333, G862, G863, G865, G866, G332, G861, G864, H540, H740 |
